# Durability and microstructure of polymer-based cement joint sealant

**DOI:** 10.1038/s41598-025-89309-1

**Published:** 2025-02-19

**Authors:** Tao Chen, Zhihang Wang, Pengfei Qin, Libin Fu, Wenjing Wang, Erlei Bai

**Affiliations:** 1 Huanghe S&T University, Zhengzhou, 450063 China; 2 Air Force Logistics University, Xuzhou, 221000 China; 3 Aviation Engineering School, Air Force Engineering University, Xi’an, 710038 China

**Keywords:** Joint sealant, Polymer, Cement, Durability, Microstructure, Engineering, Materials science

## Abstract

To promote the engineering application of polymer-based cement joint sealant (PCJS), the durability of PCJS was studied by testing the bonding, tensile and shear properties of PCJS under different service conditions. The results show that PCJS has excellent water resistance, acid/alkali corrosions resistance, UV aging resistance and low temperature resistance. The retention rate of bonding property of PCJS can achieve 85%. After water soaking, dry–wet cycle, acid/alkali corrosion, the retention rates of tensile and shear properties of PCJS can achieve 80%. After UV aging and low temperature treatment, the tensile and shear properties of PCJS are improved. After gasoline corrosion and high temperature treatment, the retention rates of tensile and shear properties of PCJS exhibit larger than 60%. The durability indexes of PCJS fulfill the technical requirements, and PCJS exhibits even more superior properties. Consequently, PCJS can be applied to joint engineering of cement concrete pavement.

## Introduction

Cement concrete pavement has the advantages of high load-bearing capacity, stable performance, long service life and low maintenance cost, and it is one of the main paving types of high grade road surface. Cement concrete pavement has been widely used in highway, urban road, airport runway and harbour road^1,2^. In order to effectively control the expansion, warping and temperature stresses of the pavement slab, and reduce the damage of the road slab caused by expansion and warping deformation, it is necessary to set a variety of joints on the pavement structure at every certain distance^3,4^. The joints should be sealed with joint sealant to maintain the smoothness and tightness of pavement and improve the durability and driving comfort of pavement^5–7^.

Joint is one of the important components of cement concrete pavement, but also one of the weakest parts, which is an important factor leading to the damage of pavement^8^. Cement concrete pavement is susceptible to various kinds of diseases such as cracking, broken plate, subsidence and misalignment^9,10^. There are many reasons for these diseases, but they are largely related to the failure of joints. And the failure of joints is largely correlated with the property of joint sealant. In the service process, the joint sealant is affected by traffic load and environmental factors, and the phenomena of stress concentration and serious aging occur easily, which is not conducive to its long-term use^11,12^. The failure of joint sealant has a great impact on the service life and service quality of pavement, and the durability of joint sealant has become one of the important bottlenecks restricting the development and application of cement concrete pavement^13,14^.

At present, common joint sealant includes asphalt, silicone, polyurethane and so on^15,16^. These materials have excellent tightness and deformation performance, but are prone to aging, cracking, debonding, water seepage and other durability concerns. The components of these joint sealants are highly sensitive to ultraviolet light and temperature, and have poor chemical stability, so they are extremely volatile and metamorphic under actual service conditions^13,17^. Engineering practice shows that these joint sealants usually has obvious shrinkage, hardening, cracking, peeling and other aging phenomena soon after use^18,19^. What is more, in some areas with large temperature differences, after a hot and cold cycle, these joint sealants break and lose stick. So it has become one of the focus in the research field of cement concrete pavement to study the joint sealant with better property and durability^20,21^. Polymer-based cement joint sealant (PCJS) is a kind of high performance composite material with two component^22^. Due to the interaction between the dehydration and film forming of polymer, and the hydration and hardening of cement, PCJS has the advantages of excellent durability and strength of cement-based material and excellent cohesibility and flexibility of polymer material^23,24^.

The current research on PCJS mainly focuses on its mix ratio design, property optimization and other aspects. Liu and Huang et al. study the effects of powder-liquid ratio and cement content on the property of PCJS, and they determine the basic mix ratio parameters of PCJS^25^. Xu et al. study the modification effects of filmforming aid, plasticizer, silane coupling agent and nano-alumina on the property of PCJS^26^. Bai et al. study the effect of carbon fiber on the property of PCJS and find that the optimal content of carbon fiber is 0.1%^27^. Wang et al. study the effects of VAE emulsion and filler on the property of PCJS, and find that the optimal content range of VAE emulsion is 30–40%, and talc powder and calcium carbonate should be used as filler^28^.

The above studies have verified the application of PCJS to cement concrete pavement joints, but there are relatively few studies on its durability^29,30^. Moreover, these studies only involve the durability of PCJS in some service conditions, such as water immersion, dry–wet cycle, gasoline corrosion, etc. In practical engineering application, PCJS faces complex and changeable service conditions, such as rain soaking, air drying, high temperature, snow, natural light irradiation, gasoline leakage, chemical corrosion of deicing and melting agents. Therefore, in order to promote the engineering application of PCJS, it is necessary to comprehensively explore the durability of PCJS in different service conditions.

Based on this, this paper studied the durability of PCJS such as water resistance, corrosion resistance, UV aging resistance and high/low temperature resistance by testing the bonding, tensile and shear properties of PCJS after water soaking, dry–wet cycle, acid/alkali/gasoline corrosion, UV aging, high/low temperature treatment and cold stretching-hot pressing. And the microstructure of PCJS under different durability test conditions was analyzed by SEM test and MIP test.

## Experiment

### Specimen preparation

**Fig. 1:**
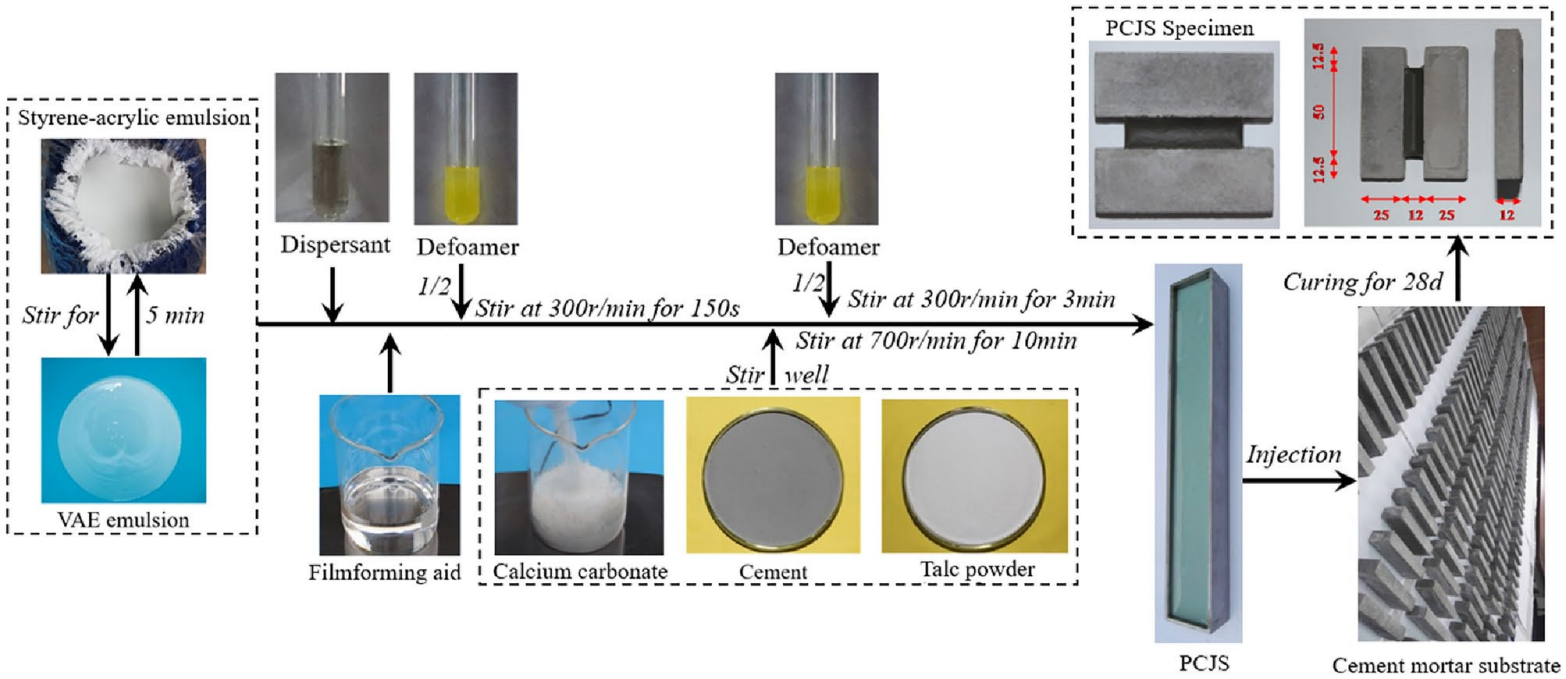
The preparation process of PCJS specimens.

Base materials, fillers and auxiliaries are used to prepare PCJS. Base materials include styrene-acrylic emulsion (Acronal S400F ap), VAE emulsion (Celvolit 1350) and cement (P∙O 42.5). Fillers include talc powder and calcium carbonate. Auxiliaries include dispersant (SN-DISPERSANT 5040), defoamer (NOPCO NXZ) and filmforming aid (DN-12). Based on previous research^22,26^, the mix ratio of PCJS is shown in Table 1, and the preparation process of PCJS specimens is shown in Fig. 1. The physical and chemical indexes of raw materials are shown in Table 2. The temperature of specimen preparation is 20 °C.

**Table 1:** The mix ratio of PCJS (unit: g/serving).

Base materials			Fillers		Auxiliaries		
Styrene-acrylic emulsion	VAE emulsion	Cement	Talc powder	Calcium carbonate	Dispersant	Defoamer	Filmforming aid
65	35	14	13	13	1.12	0.7	6

**Table 2:** The physical and chemical indexes of raw materials.

	Solid content/%	Viscosity/mPa, s	pH	*T*_*g*_/°C	MFFT/°C	Average particle size/μm	
Styrene-acrylic emulsion and VAE emulsion							
Styrene-acrylic emulsion	56 ± 1	400–1800	7.0–8.5	− 7	0	0.1	
VAE emulsion	55 ± 1	1500–5000	4.5–6.0	− 10	0	1.5	

Setting time/min		Stability	Flexural strength/MPa		Compressive strength/MPa		
Initial setting time	Final setting time		3d	28d	3d	28d	
Cement							
141	296	Qualified	6.7	9.1	32.2	54.6	

Particle size/μm	SiO_2_ content/%	MgO/%					
Talc powder							
20	60	30					

Particle size/μm	CaCO_3_ content/%						
Calcium carbonate							
30	99						

	Main component	Appearance					
Auxiliaries							
Dispersant	Polycarboxylate sodium salt	Light yellow liquid					
Defoamer	Silicone	Grey and white turbid liquid					
Filmforming aid	Texanol	Colorless clear liquid					

### Test methods

**Fig. 2:**
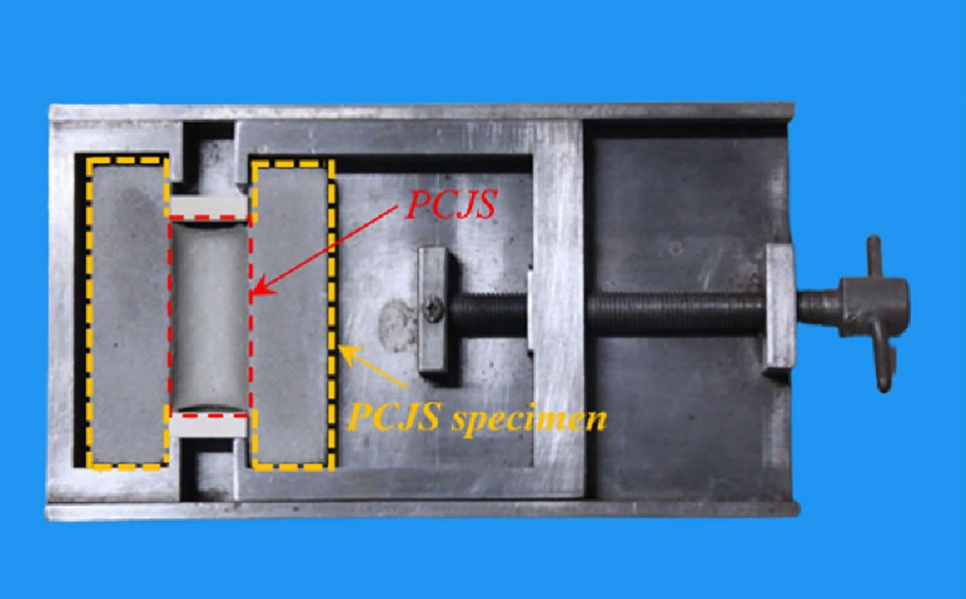
Bonding test.

Bonding test: The bonding test is carried out according to GB/T 13477.17-2017^30^. The PCJS specimen is put into the mold, as shown in Fig. 2, and then the PCJS is stretched with 60% elongation. After fixation for 24 h, the PCJS is checked if destroyed. If not destroyed, the PCJS specimen is taken out from mold, the width of PCJS is measured and the elastic recovery rate is calculated after 24 h. Each experimental group is tested in triplicate.

**Fig. 3:**
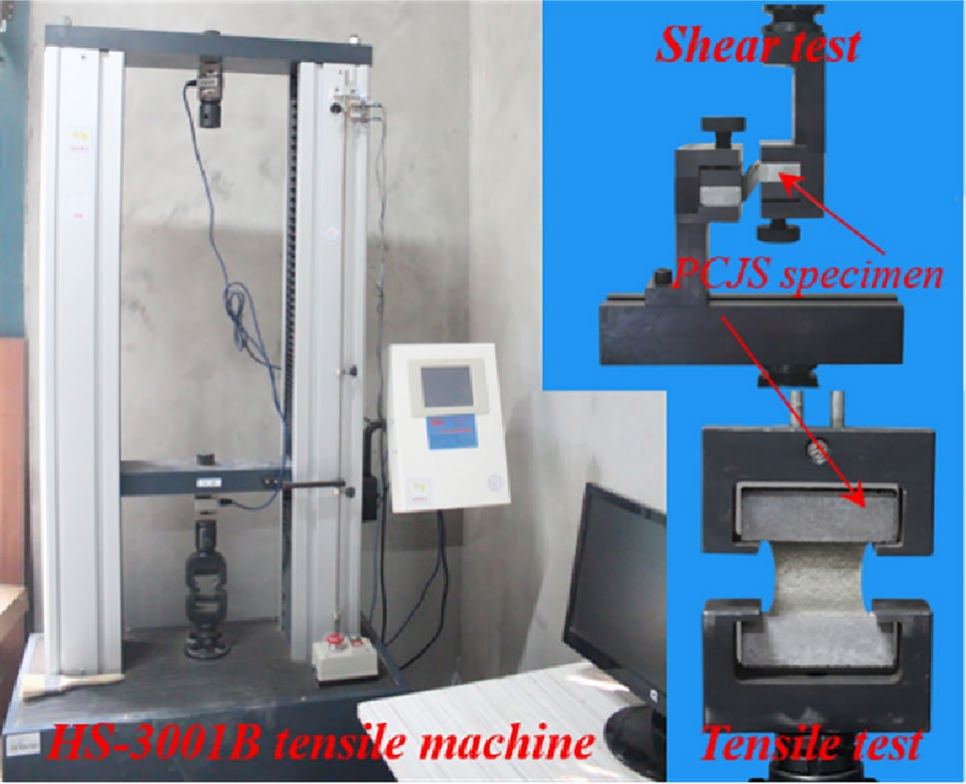
Tensile and shear tests.

Mechanical properties tests: The tensile and shear tests of PCJS specimens are carried out on the HS-3001B tensile machine according to JC/T 976-2005^31^, as shown in Fig. 3. Both the tensile and shear rates are 5 mm/min. Each experimental group is tested in triplicate.

Water soaking treatment: PCJS specimen is placed in a distilled water (23 °C) for 7 d. Dry–wet cycle treatment: PCJS specimen is placed in a distilled water (23 °C) for 1 d, then taken out and placed in a drying oven (23 °C) for 1 d. And the above steps are repeated 5 times. The properties of PCJS specimen are tested after water soaking and dry–wet cycle.

Acid/alkali/gasoline corrosion treatment: PCJS specimen is immersed in H_2_SO_4_ solution (pH = 1, 23 °C), NaOH solution (pH = 13, 23 °C) and gasoline (23 °C) for 7 d, respectively. The properties of PCJS specimen are tested after acid/alkali/gasoline corrosion.

UV aging treatment: PCJS specimen is exposed to ultraviolet aging tester for 7 d and the wavelength of the irradiation is 315 nm ~ 400 nm. The properties of PCJS specimen are tested after UV aging.

High/low temperature treatment: PCJS specimen is placed in the high-low temperature test chamber, and treated at a high temperature (70 °C) and a low temperature (− 20 °C) for 7 d respectively. Cold stretching-hot pressing treatment: PCJS specimen is placed in the chamber (− 20 °C) for 1 d after stretching, and the elongation of PCJS was 25%. Then, PCJS specimen is taken out and compressed, and the compression of PCJS is 25%. After compression, PCJS specimen is placed in the chamber (70 °C) for 1 d. And the above steps are repeated 5 times.

The bonding and mechanical properties of PCJS specimen are tested after high/low temperature treatment, and the bonding property of PCJS specimen is tested after cold stretching-hot pressing treatment. The PCJS specimen without any treatment of the durability test conditions is used as the control group.

SEM and MIP tests: The scanning electron microscope (SEM) is used to characterize the morphology of PCJS. Mercury intrusion porosimetry (MIP) is employed to characterize the pore structure of PCJS.

## Results and discussion

### Water resistance

**Fig. 4:**
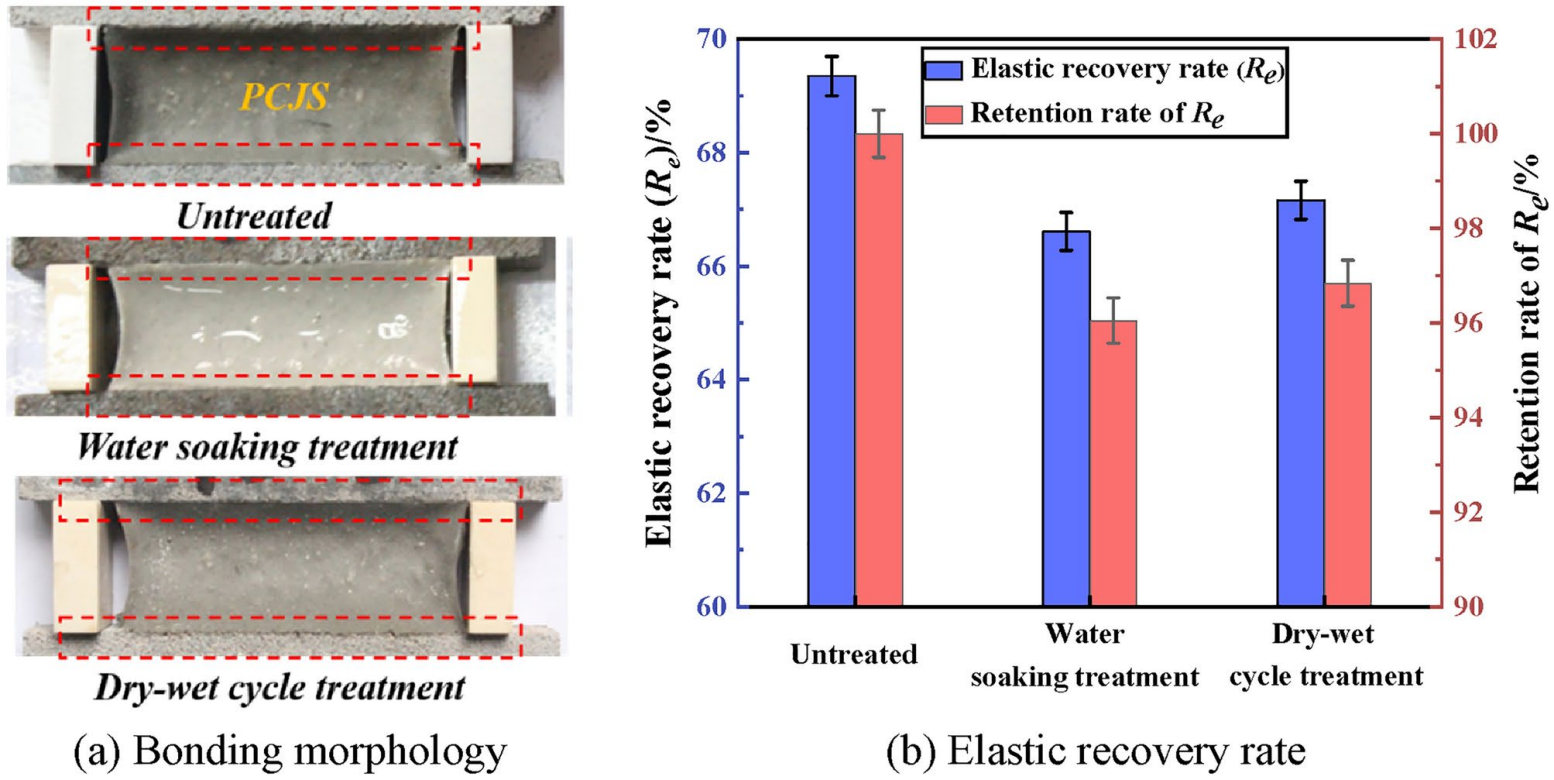
The bonding property of PCJS after water soaking and dry–wet cycle.

The bonding property of PCJS after water soaking and dry–wet cycle is shown in Fig. 4. From Fig. 4a, no damage occurs inside PCJS, and PCJS does not debond with cement mortar substrate after water soaking and dry–wet cycle. From Fig. 4b, the elastic recovery rate (*R*_*e*_) of PCJS decreases after water soaking and dry–wet cycle, but the *R*_*e*_ of PCJS is larger than 60%. The retention rates of *R*_*e*_ of PCJS are 96.05% and 96.84%, respectively, after water soaking and dry–wet cycle.

**Fig. 5:**
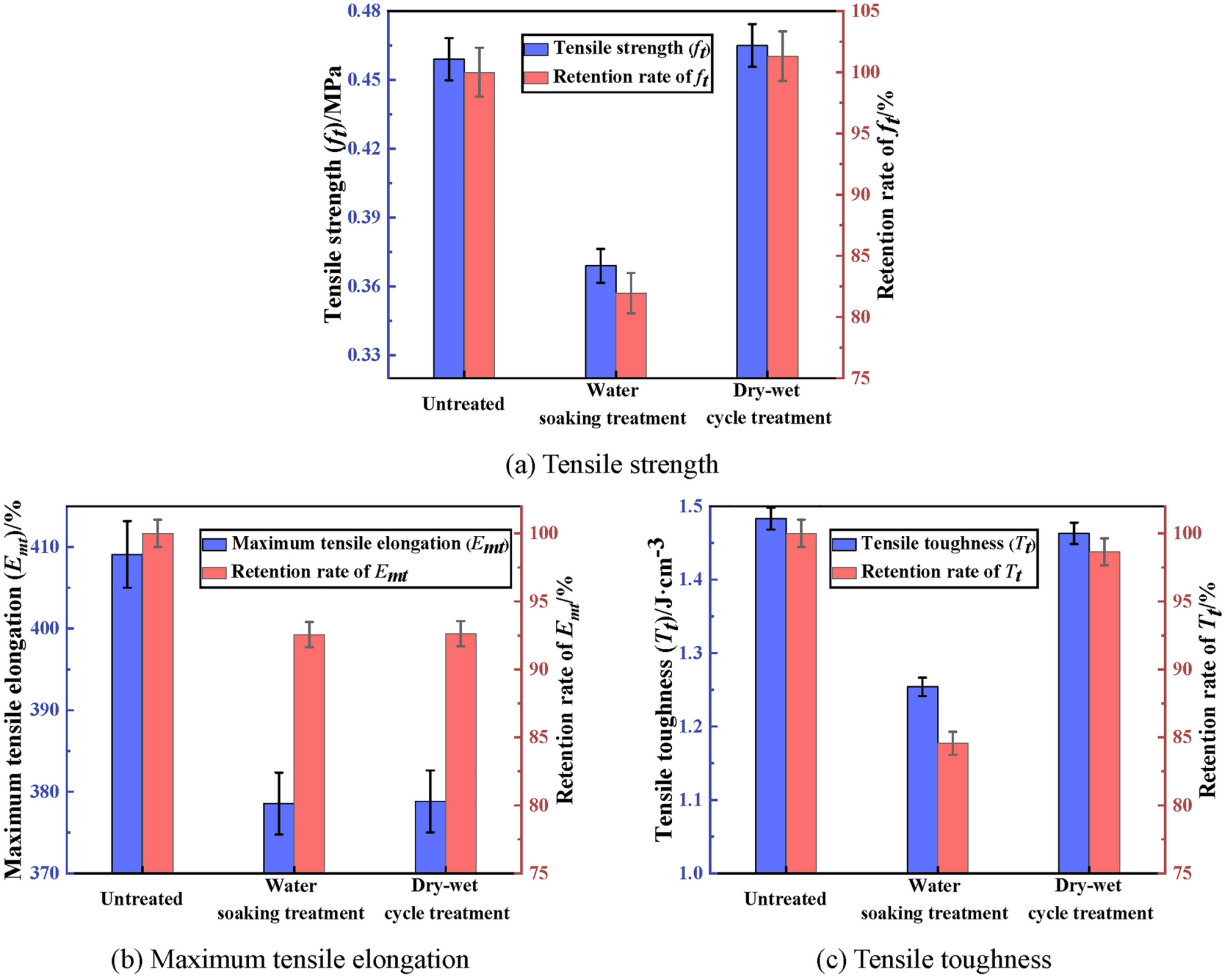
The tensile property of PCJS after water soaking and dry–wet cycle.

The tensile property of PCJS after water soaking and dry–wet cycle are shown in Fig. 5. From Fig. 5, the tensile property indexes of PCJS decrease after water soaking. The tensile strength (*f*_*t*_) of PCJS increases, while the maximum tensile elongation (*E*_*mt*_) and tensile toughness (*T*_*t*_) decrease. After dry–wet cycle, the *f*_*t*_ of PCJS can achieve 0.465 MPa. The retention rates of the tensile property indexes of PCJS are larger than 80% after water soaking and dry–wet cycle. Especially for the *E*_*mt*_, the retention rate is larger than 90%. The retention rates of *f*_*t*_ of PCJS are 81.96% and 101.31%, and the retention rates of *T*_*t*_ are 84.56% and 98.65% after water soaking and dry–wet cycle. The retention rates of tensile property indexes of PCJS after dry–wet cycle are larger than that after water soaking.

**Fig. 6:**
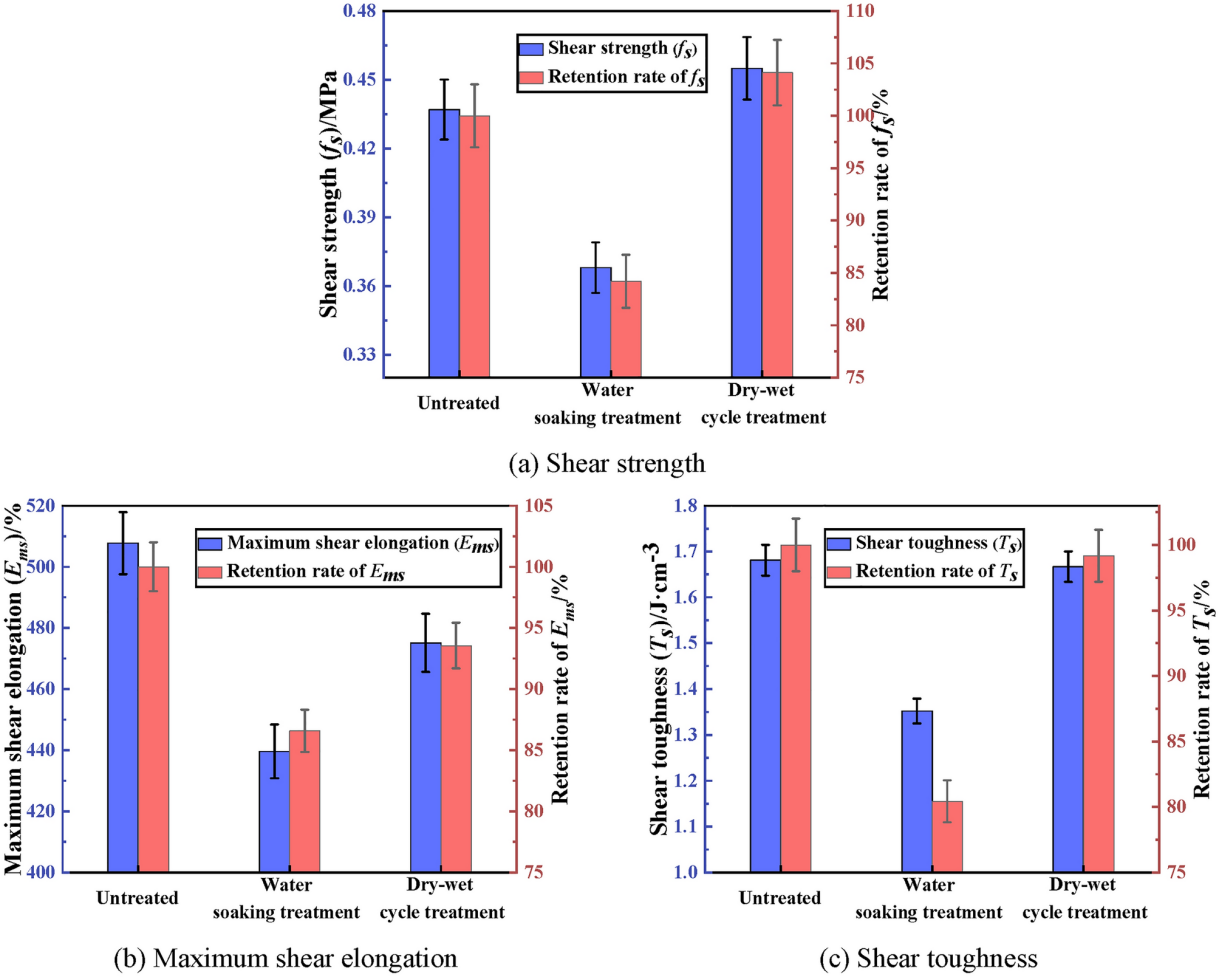
The shear property of PCJS after water soaking and dry–wet cycle.

The shear property of PCJS after water soaking and dry–wet cycle is shown in Fig. 6. From Fig. 6, the variation of shear property indexes of PCJS is consistent with that of tensile property indexes after water soaking and dry–wet cycle. The shear property indexes of PCJS decrease after water soaking. The shear strength (*f*_*s*_) of PCJS increases, while the maximum shear elongation (*E*_*ms*_) and shear toughness (*T*_*s*_) decrease. The retention rates of shear property indexes of PCJS are larger than 80% after water soaking. The retention rate of shear property indexes of PCJS are larger than 90% after dry–wet cycle. Especially for the *f*_*s*_ and *T*_*s*_, the retention rates are 104.12% and 99.17%, respectively. The retention rates of shear property indexes of PCJS after dry–wet cycle are larger than that after water soaking. From the above analysis, it is known that PCJS still shows excellent bonding, tensile and shear properties after water soaking and dry–wet cycle.

There are two kinds of effect of water on joint sealant. One is plasticization. Water can form hydrogen bonds with polar groups in joint sealant, which weakens the hydrogen bonds between polymer molecules and deteriorates the properties of joint sealant^31^. The other is hydrolysis. Water reacts with the hydrolyzed groups (such as ester, carboxyl, hydroxyl, etc.) in polymer molecules to break the polymer molecular chain^30^. The main components of PCJS are styrene-acrylic emulsion and VAE emulsion. The phenyl ring and aliphatic side-chain in the two polymers are hydrophobic, which can effectively prevent water from entering the PCJS and weaken the plasticization and hydrolysis of water^33^. Therefore, PCJS has excellent water resistance. During the dry–wet cycle, the unhydrated cement inside PCJS has a secondary hydration reaction with the infiltrated water, so the tensile and shear strength of PCJS increase^34^. In the process of water soaking, secondary hydration of cement also exists inside PCJS, but the deterioration of PCJS caused by water is more obvious such as plasticization and hydrolysis. Therefore, the retention rates of property indexes of PCJS after dry–wet cycle are larger than that after water soaking.

### Corrosion resistance

**Fig. 7:**
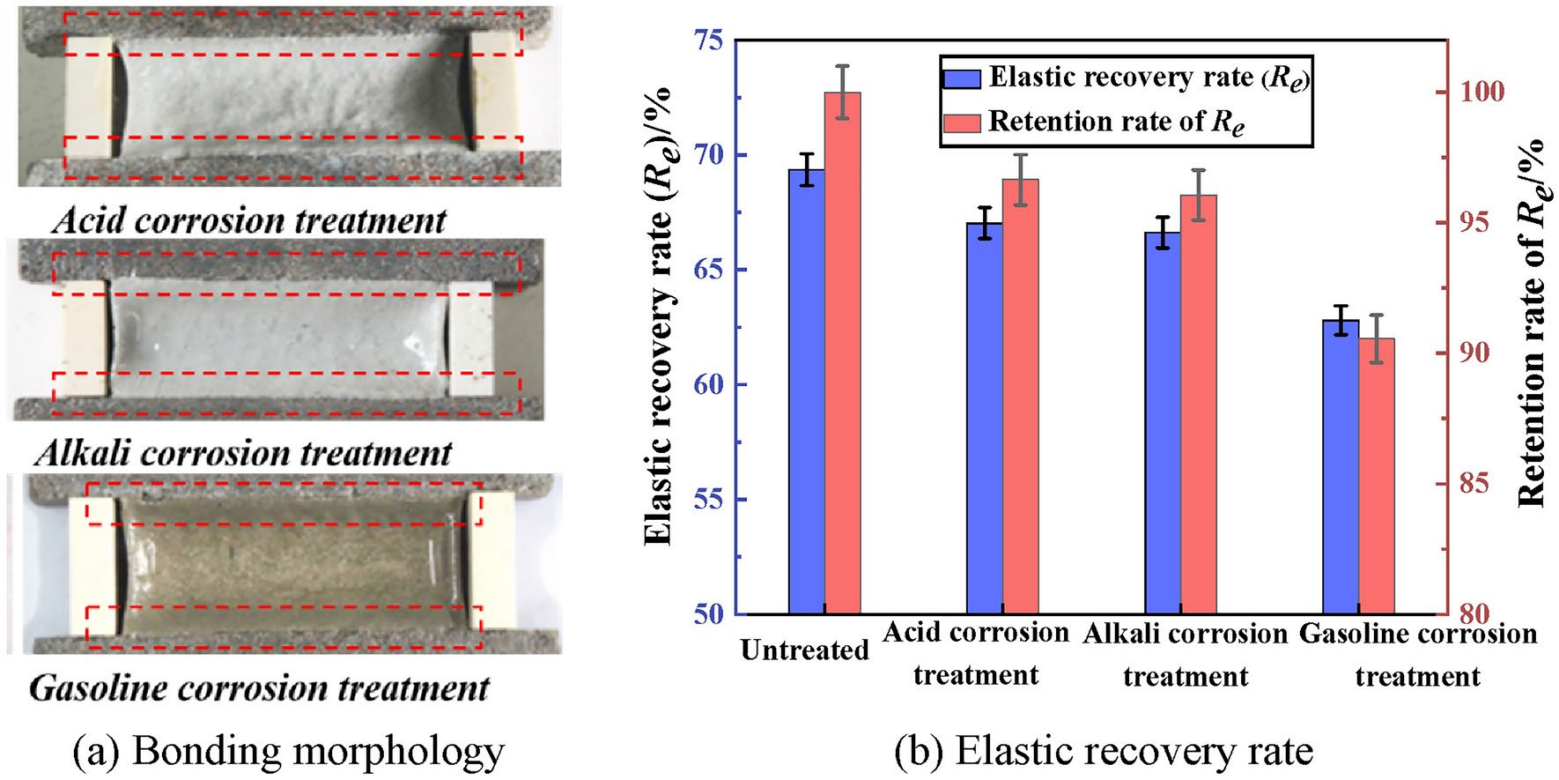
The bonding property of PCJS after acid/alkali/gasoline corrosion.

The bonding property of PCJS after acid/alkali/gasoline corrosion is shown in Fig. 7. From Fig. 7, after acid/alkali/gasoline corrosion, PCJS does not incur internal damage and debonding failure, and the elastic recovery (*R*_*e*_) rate of PCJS is larger than 60%. After acid/alkali/gasoline corrosion, the *R*_*e*_ of PCJS decreases, but the retention rate of *R*_*e*_ is larger than 90%. After gasoline corrosion, the retention rate of *R*_*e*_ of PCJS is the minimum, which is 90.56%. This shows that acid, alkali and gasoline corrosion have minor impact on the bonding property of PCJS.

**Fig. 8:**
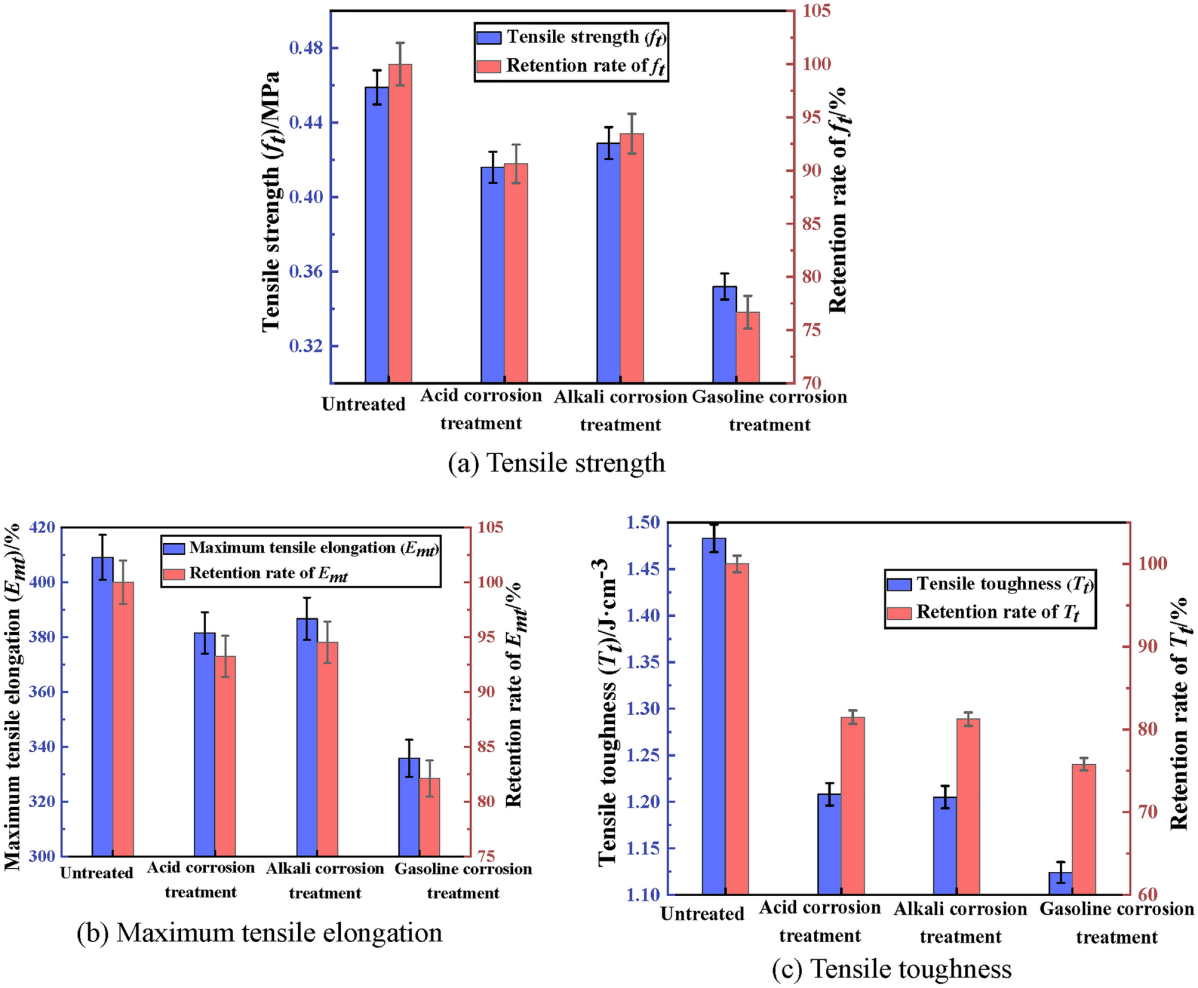
The tensile property of PCJS after acid/alkali/gasoline corrosion.

The tensile property of PCJS after acid/alkali/gasoline corrosion is shown in Fig. 8. From Fig. 8, the tensile property indexes of PCJS decrease after acid/alkali/gasoline corrosion. After gasoline corrosion, the tensile property indexes of PCJS are the minimum, and the retention rates of tensile strength (*f*_*t*_), maximum tensile elongation (*E*_*mt*_) and tensile toughness (*T*_*t*_) are 76.69%, 82.10% and 75.79%, respectively. After acid and alkali corrosion, the retention rates of *f*_*t*_ and *E*_*mt*_ of PCJS are larger than 90%, and the retention rate of *T*_*t*_ is larger than 80%. The deterioration effect of gasoline corrosion on the tensile property of PCJS is stronger than that of acid/alkali corrosion. Taken as a whole, the retention rates of tensile property indexes of PCJS are still larger than 75% after acid/alkali/gasoline corrosion.

**Fig. 9:**
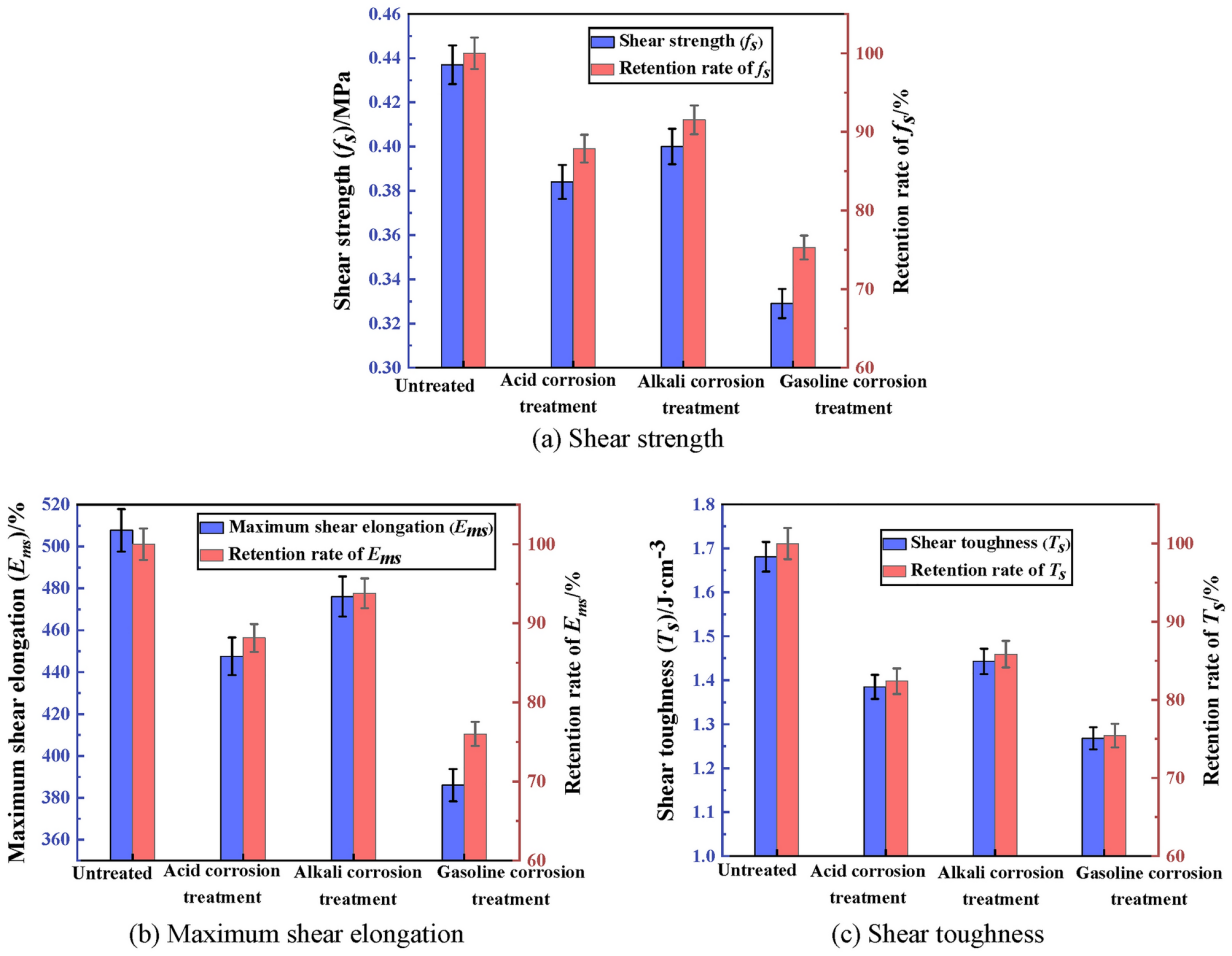
The shear property of PCJS after acid/alkali/gasoline corrosion.

The shear property of PCJS after acid/alkali/gasoline corrosion is shown in Fig. 9. From Fig. 9, after acid/alkali/gasoline corrosion, the shear strength (*f*_*s*_), maximum shear elongation (*E*_*ms*_) and shear toughness (*T*_*s*_) of PCJS decrease. After acid corrosion, the retention rates of shear property indexes of PCJS are larger than 80%. After alkali corrosion, the retention rates of shear property indexes of PCJS are larger than 85%. After gasoline corrosion, although the shear property of PCJS is the worst, the retention rates of the shear property indexes of PCJS are still larger than 75%. From the above analysis, it is known that acid, alkali and gasoline all deteriorate the bonding, tensile and shear properties of PCJS to varying degrees, and the deterioration of acid and alkali on the properties of PCJS is less. The acid and alkali corrosion resistance of PCJS are better. Gasoline has the most significant effect on the deterioration of PCJS properties, but the retention rates of property indexes of PCJS are larger than 75%.

Since polymers (styrene-acrylic emulsion and VAE emulsion) have excellent acid and alkaline resistance, the synergistic effect of acid/alkali and water on the properties of PCJS is similar to that of water soaking. So PCJS can still maintain good properties after being corroded by acid and alkali solution^35^. In addition, by comparing the results of the effect of water soaking on the properties of PCJS in Section "Water resistance", we can find that the deterioration degree of acid/alkali corrosion on the properties of PCJS is less than that of water soaking. Due to the hydrophobicity of polymer molecules, when soaked in acid and alkali solutions, less water penetrates into PCJS, and the plasticization of water weakens^29^. Meanwhile, acid and alkali also delay the hydrolysis of water. Therefore, the properties of PCJS after acid/alkali corrosion is better than that after water soaking. After gasoline corrosion, gasoline is easy to penetrate into the PCJS. The infiltrated gasoline makes the intertwined polymer molecular chains swell and weakens the intermolecular forces and, which results in a significantly decrease in the properties of PCJS^30^.

### UV aging resistance

**Fig. 10:**
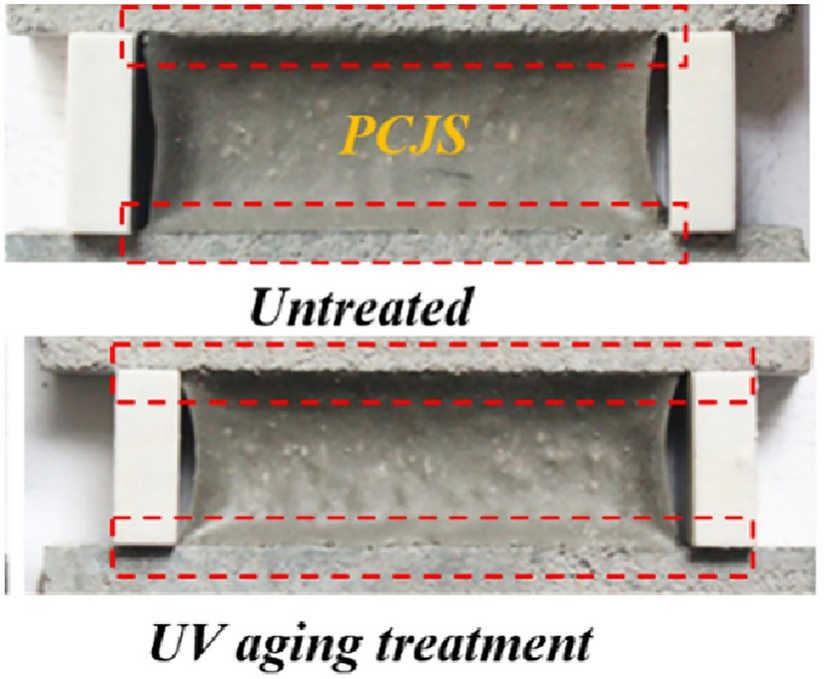
Bonding morphology.

The bonding morphology of PCJS after UV aging is shown in Fig. 10, and the properties indexes are shown in Table 3. From Fig. 10, no damage occurs inside PCJS and PCJS has good bond morphology after UV aging. From Table 3, the elastic recovery rate of PCJS increases by 4.14% after UV aging. UV aging does not impact the binding property of PCJS, and even has beneficial effect on it. After UV aging, the strength and toughness of PCJS increase, and the maximum elongation decreases. The tensile and shear strength of PCJS increase by more than 50%, and the tensile and shear toughness increase by 40.32% and 34.09%, respectively. After UV aging, the retention rates of maximum tensile/shear elongation of PCJS are larger than 85%. It can be seen that PCJS has excellent UV aging resistance, and most of the property indexes of PCJS increase after UV aging. The ultraviolet irradiation energy (299.4 ~ 399.0 kJ/mol) is larger than the bond energy of polymer molecular chain (40 ~ 100 kJ/mol), so under the ultraviolet irradiation, the polymer molecules are excited, and the photooxygen reaction occurs^37^. The photooxygen reaction leads to cross-linking between polymer molecular chains and some cement hydration products, which enhances the integrity of PCJS. The cross-linking also makes the rigidity of PCJS enhanced^38^. Therefore, the external force required by PCJS in tensile or shear failure increases, while the deformation decreases.

**Table 3:** The properties indexes of PCJS after UV aging.

	Elastic recovery rate/%	Tensile strength/MPa	Maximum tensile elongation/%	Tensile toughness/J cm^−3^	Shear strength/MPa	Maximum shear elongation/%	Shear toughness/J cm^−3^
Untreated	69.35	0.459	409.09	1.483	0.437	507.73	1.681
After UV aging	72.22	0.702	358.12	2.081	0.687	449.54	2.254
Retention rates/%	104.14	152.94	87.54	140.32	157.21	88.54	134.09

### High/low temperature resistance

**Fig. 11:**
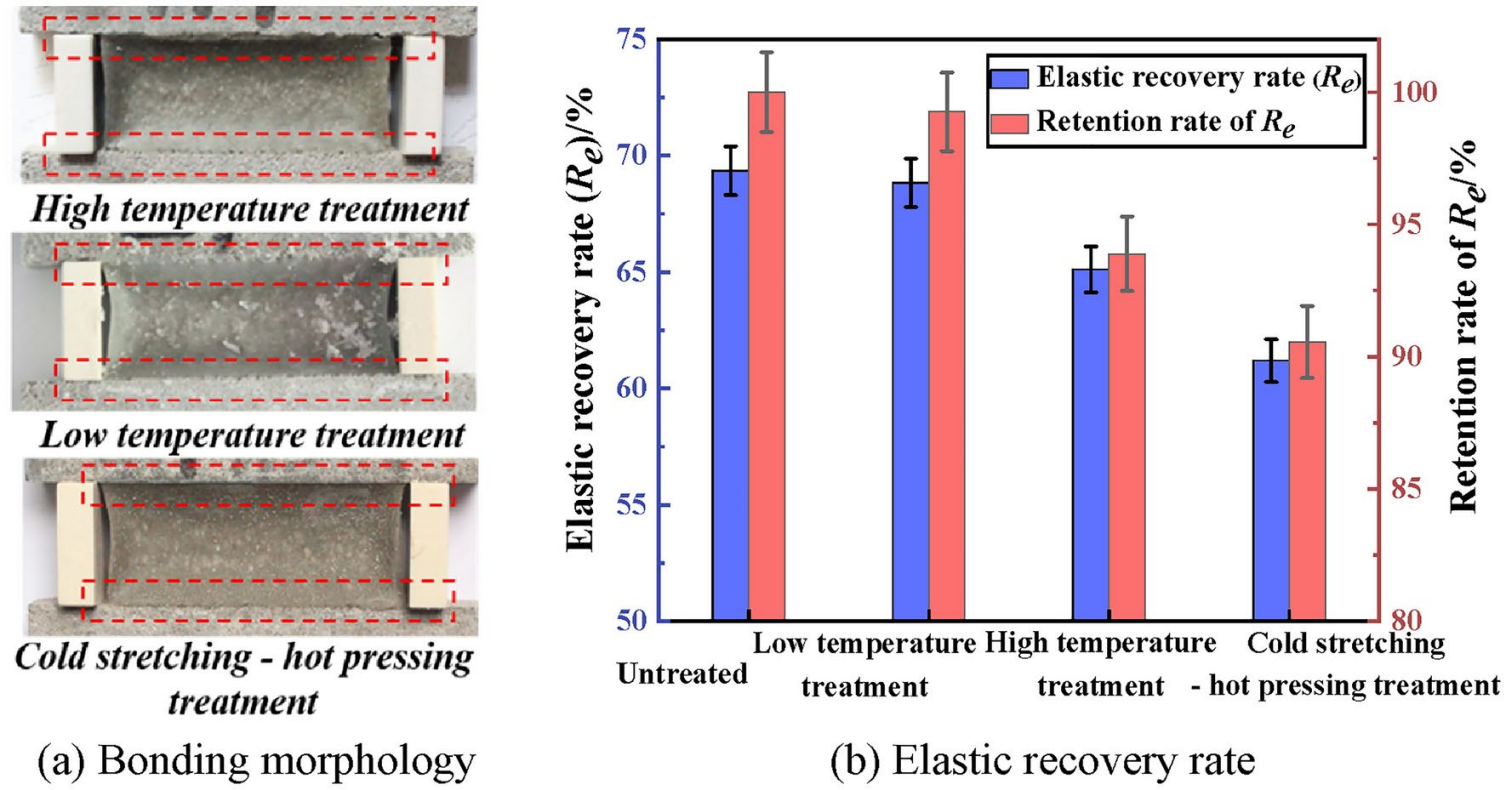
The bonding property of PCJS after high/low temperature and cold stretching-hot pressing treatments.

The bonding property of PCJS after high/low temperature and cold stretching-hot pressing treatments is shown in Fig. 11. From Fig. 11, after high/low temperature and cold stretching-hot pressing treatments, PCJS does not damage or debond, and the elastic recovery rate (*R*_*e*_) is larger than 60%, which satisfies the requirement of the bonding property of the joint sealant. After high/low temperature and cold stretching-hot pressing treatments, the retention rates of *R*_*e*_ of PCJS are larger than 85%. The effect of low temperature treatment on the* R*_*e*_ of PCJS is very small, and its retention rate is 99.25%.

**Fig. 12:**
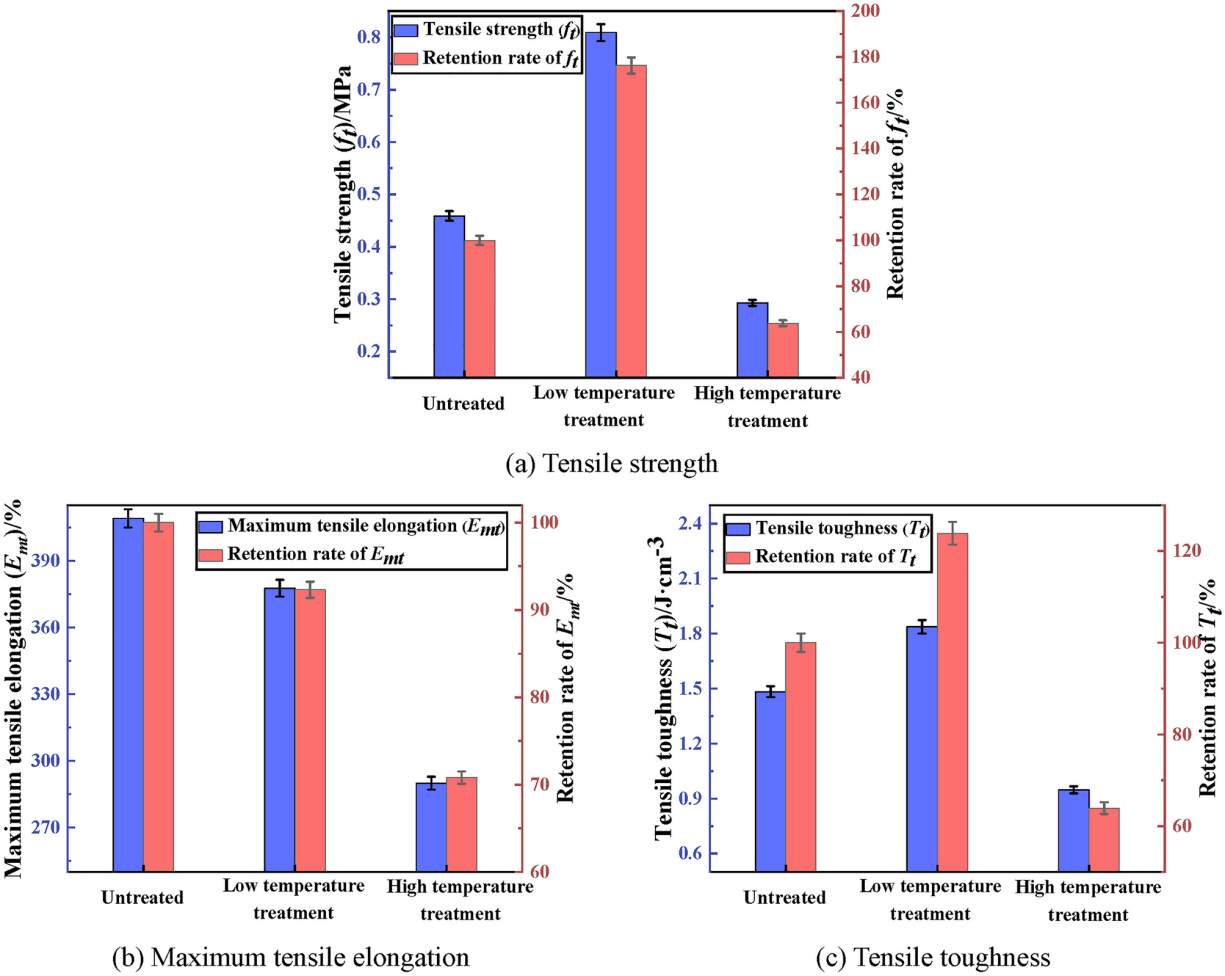
The tensile property of PCJS after high/low temperature treatment.

The tensile property of PCJS after high/low temperature treatment is shown in Fig. 12. From Fig. 12, after low temperature treatment, the tensile strength (*f*_*t*_) and toughness (*T*_*t*_) of PCJS increase by 76.25% and 23.87%, respectively. The maximum tensile elongation (*E*_*mt*_) decreases, but its retention rate is 92.31%. PCJS still shows excellent tensile property after low temperature treatment. After high temperature treatment, the tensile property indexes of PCJS decrease. The retention rates of *f*_*t*_ and *T*_*t*_ are larger than 60%, and the retention rate of *E*_*mt*_ is larger than 70%. After high temperature treatment, the *E*_*mt*_ of PCJS is still 289.93%.

**Fig. 13:**
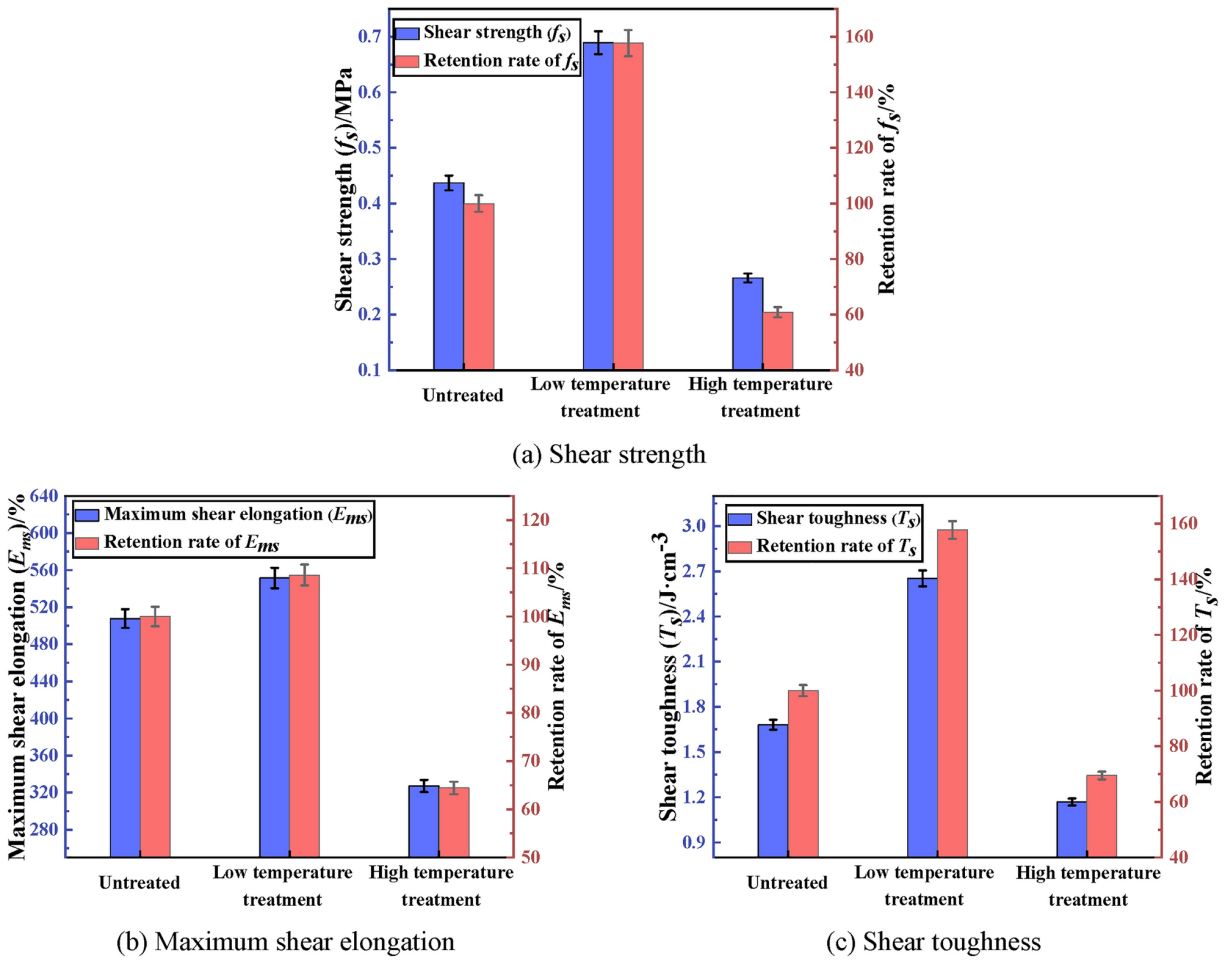
The shear property of PCJS after high/low temperature treatment.

The shear property of PCJS after high/low temperature treatment is shown in Fig. 13. From Fig. 13, after low temperature treatment, the shear property of PCJS improves, and the shear property indexes increase. The shear strength and toughness increase by 57.67% and 57.76%, and the maximum shear elongation increases by 8.61%. After high temperature treatment, the shear property of PCJS decreases. But the retention rates of shear property indexes are all larger than 60%. The effect of high temperature treatment on the properties of PCJS is comparatively large, but the retention rates of properties indexes are all larger than 60%.

Both styrene-acrylic emulsion and VAE emulsion have excellent low temperature resistance. In addition, the energy in low temperature environment is less, and the thermal motion of polymer molecules is severely inhibited. So the polymer molecular chains are in the freezing and condensation state and the intermolecular force increases^39^. Therefore, the properties of PCJS improve after low temperature treatment. At high temperature, the thermal motion of molecules is enhanced, which leads to the degradation and fracture of polymer molecular chains^29,30^. Therefore, the properties of PCJS decrease after high temperature treatment.

### Durability analysis

At present, there are relatively few standards for durability indexes of joint sealant^29,30^. And in these standards, only the requirements for the bonding property of joint sealant after water soaking, high/low temperature treatment (cold stretching-hot pressing) and gasoline corrosion are involved. And there are no specific requirements for durability indexes such as UV aging resistance and acid/alkali corrosion resistance. Wang et al. and Yuan et al.^40,41^ combined with the development situation of joint sealant, made a comprehensive comparative analysis of relevant standards, and put forward the technical requirements that joint sealant should meet.Therefore, we analyzes the durability of PCJS by referring to the durability indexes in reference^40,41^. The technical requirements in the reference^40,41^ for the water resistance, gasoline corrosion resistance and high/low temperature resistance of the joint sealant are listed in Table 4. The durability test results of PCJS are shown in Table 5.

**Table 4:** Technical requirements for the durability of joint sealant.

Property	Technical requirements
Water resistance	Stretch with 60% elongation after water soaking for 24 h, no failure
Gasoline corrosion resistance	Stretch with 60% elongation after gasoline corrosion for 24 h, no failure
High/low temperature resistance	Stretch with 60% elongation after cold stretching-hot pressing (− 20 °C × 24 h, 70 °C × 24 h) treatment, no failure

**Table 5:** Durability test results of PCJS.

Property	Durability test condition of PCJS	Test result
Water resistance	Stretch with 60% elongation after water soaking for 7 days	No failure
	Stretch with 60% elongation after 5 dry–wet cycles	
Gasoline corrosion resistance	Stretch with 60% elongation after gasoline corrosion for 7 days	No failure
Acid/alkali corrosion resistance	Stretch with 60% elongation after acid/alkali corrosion for 7 days	No failure
UV aging resistance	Stretch with 60% elongation after UV aging for 7 days	No failure
High/low temperature resistance	Stretch with 60% elongation after 5 cold stretching-hot pressing (− 20 °C × 24 h, 70 °C × 24 h) treatments	No failure
	Stretch with 60% elongation after high temperature treatment (70 °C × 7 days)	
	Stretch with 60% elongation after low temperature treatment (− 20 °C × 7 days)	

From Table 5, PCJS can still keep excellent bonding property without damage after water soakingfor 7 d, gasoline corrosion for 7d and 5 cold stretching-hot pressing treatments. In addition, PCJS can still keep excellent bonding property after other durability test conditions, such as 5 dry- wet cycles, acid/alkali corrosion for 7d, UV aging for 7d and high/low temperature treatment. In terms of the elastic recovery rate, tensile and shear properties of PCJS, no obvious deterioration of the properties of PCJS is found in the other durability test conditions, except that gasoline corrosion and high temperature treatment for 7 d significantly weakens the properties of PCJS. After gasoline corrosion for 7 d, the retention rates of properties indexes of PCJS are larger than 75%. After high temperature treatment for 7 d, the retention rates of properties indexes of PCJS are larger than 60%. Therefore, the above test results show that PCJS has excellent durability. The comparison of tensile strength retention rate of PCJS with other materials is shown in Table 6. From Table 6, compared with other materials, PCJS has better durability. Overall, the high temperature resistance of PCJS needs to be further improved.

**Table 6:** Comparison of tensile strength retention rate of PCJS with other materials.

Materials	Water resistance		Corrosion resistance			UV aging resistance	High/low temperature resistance	
	Water soaking	Dry–wet cycle	Acid corrosion	Alkali corrosion	Gasoline corrosion	UV aging	High temperature treatment	Low temperature treatment
PCJS	81.96%	101.31%	90.63%	93.46%	76.69%	152.94%	63.83%	176.25%
Modified asphalt^42^	–	91.10%	–	41.80%	–	–	–	87.80%
Silicone^43^	–	–	–	–	–	–	–	94.42%
Polyurethane^44^	–	–	68.02%	83.74%	–	–	–	–
Polyurethane^45^	–	–	–	–	–	98.93%	–	–
Silicone^46^	75.13%	–	–	–	–	–	–	–
Polysulfide^47^	–	–	–	–	–	–	40.60%	–
MS joint sealant^48^	–	–	–	–	–	86.00%	–	–
Organosilicon^49^	–	–	–	–	–	–	61.00%	–

## Microstructure

### Micromorphology

**Fig. 14:**
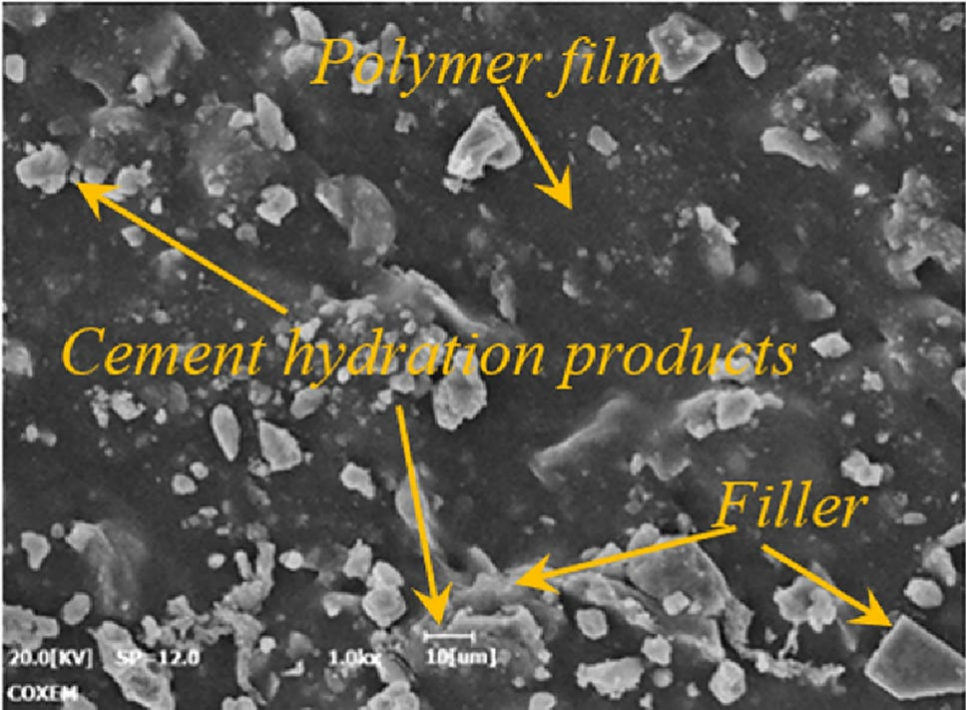
The surface morphology of PCJS.

In the preparation process of PCJS, the polymer (styrene-acrylic emulsion and VAE emulsion) accounts for the major body of PCJS, and the content of cement and filler is relatively small. The polymer dehydrates to form the film structure, and the continuous polymer film structure constitutes the matrix of PCJS. Hydration reaction occurs between cement and water, and the cement hydration products and fillers are dispersed in the matrix (as shown in Fig. 17), which act as reinforcement and filling (Fig. 14).

**Fig. 15:**
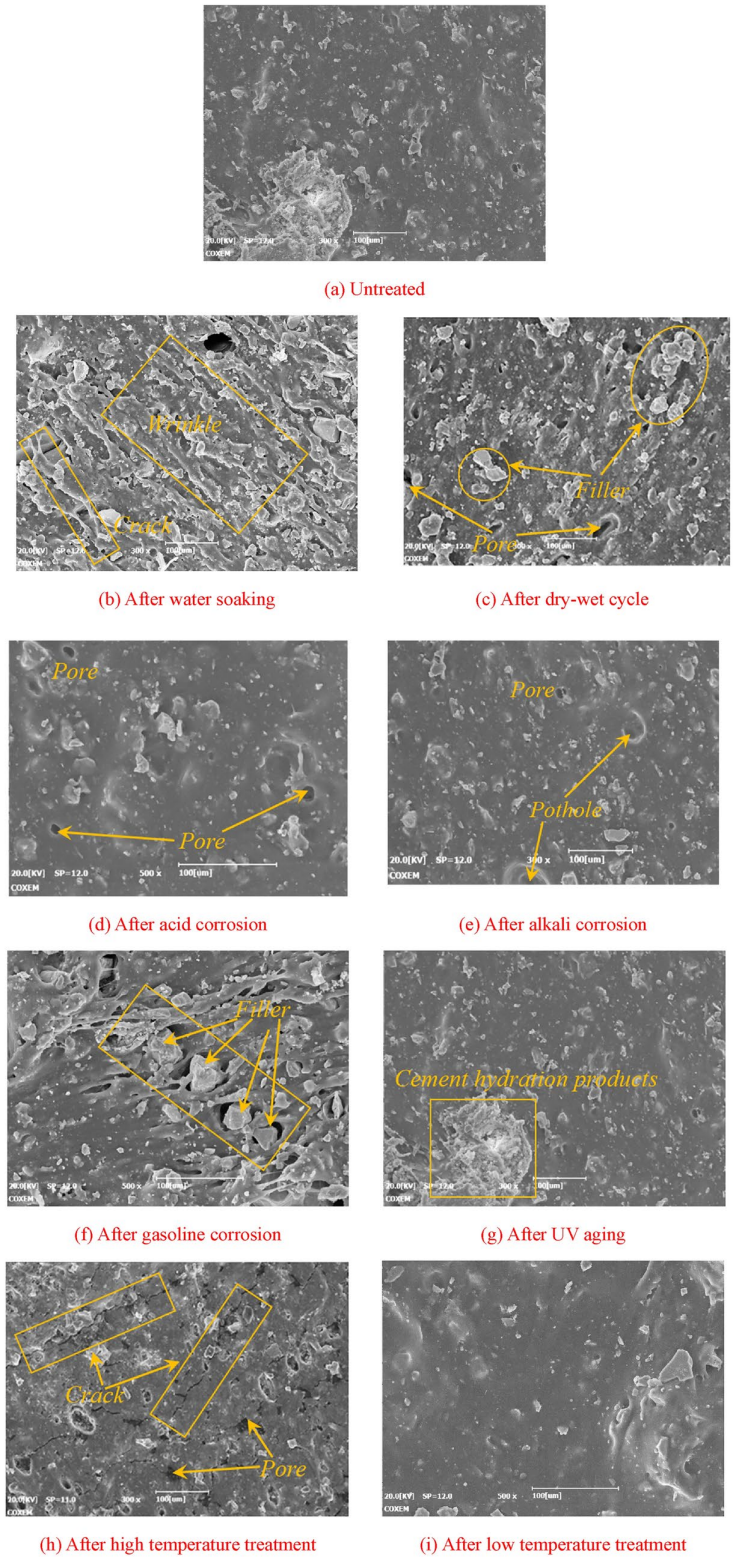
The surface morphology of PCJS with different service conditions.

The surface morphology of PCJS with different service conditions is shown in Fig. 15. From Fig. 15a, the polymer film in untreated PCJS remains intact and free of defects. From Fig. 15b, the polymer film in PCJS shows obvious strip folds and a few cracks after water soaking, but the cracks do not throughout the entire polymer film. From Fig. 15c, a small amount of the filler is removed from the polymer film matrix and a few pores appear in the matrix after dry–wet cycle. The polymer film structure keeps continuous and PCJS shows excellent water resistance. From Fig. 15d–e, only a few pores and pits appear in the polymer film matrix and no obvious defects appear after acid/alkali corrosion. PCJS shows excellent acid/alkali corrosion resistance. From Fig. 15f, the polymer film matrix near the filler is destroyed obviously, and the filler is almost completely separated from the polymer film matrix after gasoline corrosion. Gasoline corrosion obviously destroys the microstructure of PCJS. From Fig. 15g, the cross-linking between cement hydration products and polymer film structure enhances after UV aging, and the integrity of PCJS improves^37^. From Fig. 15h, a large number of cracks and pores appear in the polymer film matrix after high temperature treatment. From Fig. 15i, the morphology of PCJS does not change obviously after low temperature treatment. From the micromorphology of PCJS, except for gasoline corrosion and high temperature treatment, the polymer film matrix of PCJS under other durability test conditions has not been significantly damaged and is relatively complete, and a continuous polymer film structure is maintained inside PCJS.

### Pore structure

**Fig. 16:**
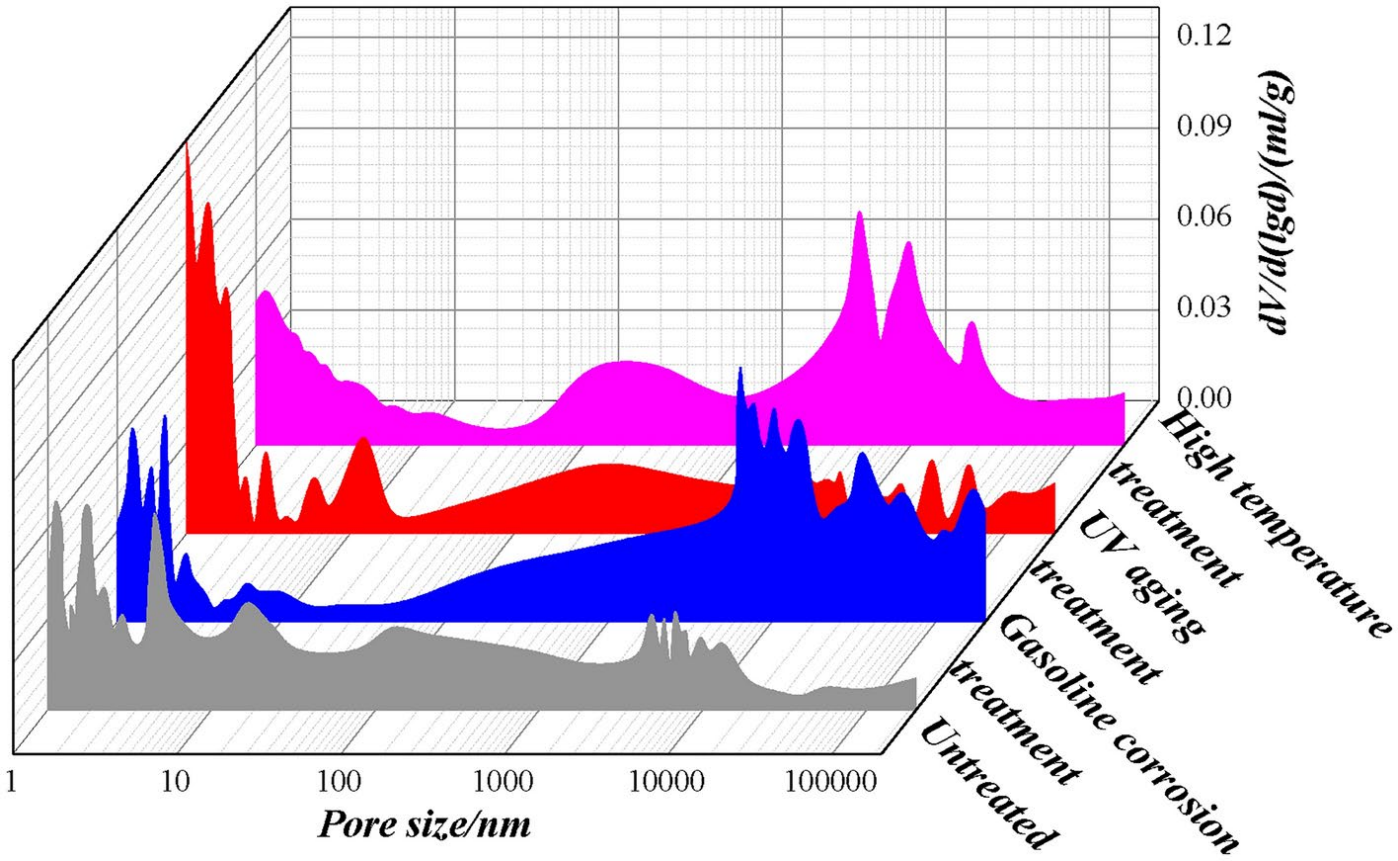
The differential curve of pore size distribution PCJS.

**Fig. 17:**
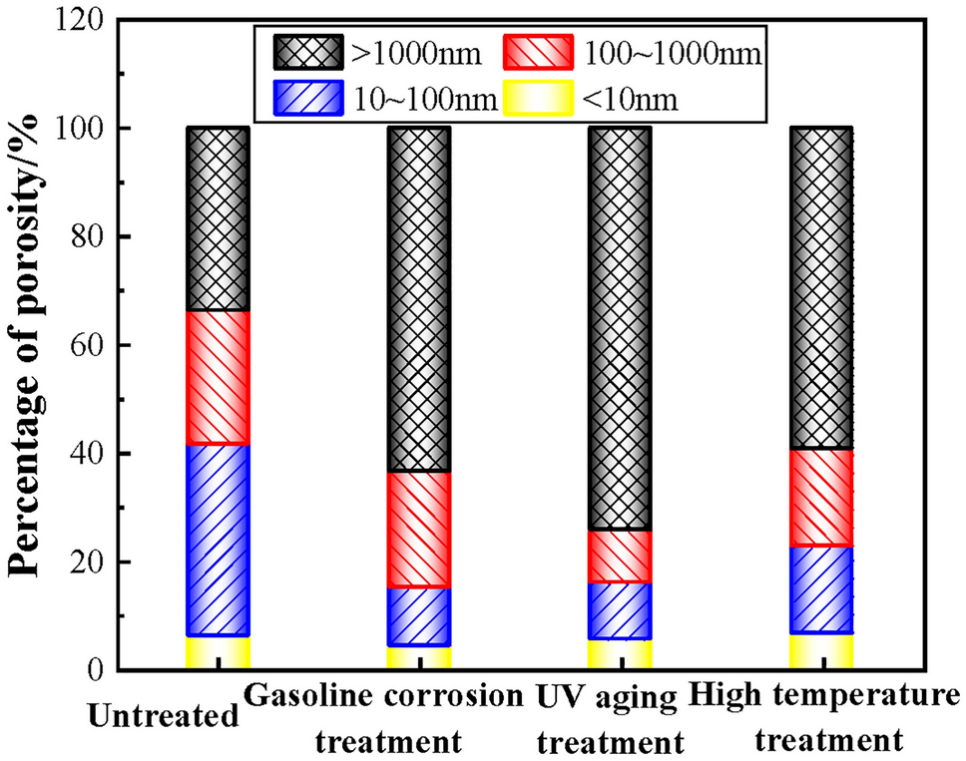
The percentage of porosity of PCJS.

The pore structure parameters of PCJS are shown in Table 7, the differential curve of pore size distribution is shown in Fig. 16, and the percentage of porosity is shown in Fig. 17. From Table 7, Figs. 16 and 17, the total porosity and average pore size of PCJS increase after gasoline corrosion and high temperature treatment. After gasoline corrosion, the total porosity and average pore size of PCJS increase by 44.61% and 56.79%, respectively. After high temperature treatment, the total porosity and average pore size of PCJS increased by 28.92% and 80.63%, respectively. After UV aging, the total porosity of PCJS decreases by 17.80% and the average pore size increases by 71.99%. After gasoline corrosion, UV aging and high temperature treatment, the pore size distribution of PCJS shifts towards macropore, and the percentage of macropore (> 1000 nm) increases, while the percentage of small pore (< 100 nm) decreases. After gasoline corrosion, UV aging and high temperature treatment, the percentage of macroporous PCJS are 63.53%, 74.29% and 59.31%, respectively. After UV aging, the cross-link reaction between polymer molecular chains and cement hydration products fills some small pores, which results in a decrease in the total porosity of PCJS and an increase in the percentage of macropore. The pore size of PCJS increases and pore structure deteriorates after gasoline corrosion and high temperature treatment.

**Table 7:** The pore structure parameters of PCJS.

Durability test condition	Total porosity/(mL/g)	Average pore size/nm	Porosit /(mL/g)			
			< 10 nm	10 ~ 100 nm	100 ~ 1000 nm	> 1000 nm
Untreated	0.0854	48.16	0.0052	0.0303	0.0211	0.0288
After gasoline corrosion	0.1235	75.51	0.0052	0.0134	0.0265	0.0785
After UV aging	0.0702	82.83	0.0038	0.0073	0.0069	0.0522
After high temperature treatment	0.1101	86.99	0.0072	0.0178	0.0199	0.0653

## Conclusion

The properties of PCJS under different durability test conditions are tested by bonding, tensile and shear tests, and the durability of PCJS is studied. The microstructure of PCJS is analyzed by SEM and MIP tests. The main conclusions are as follows.

1. PCJS has excellent durability. PCJS shows excellent water resistance, acid/alkali corrosion resistance, UV aging resistance and low temperature resistance. The durability indexes of PCJS fulfill the technical requirements, and PCJS exhibits even more superior properties.
2. The retention rate of bonding property of PCJS can achieve 85% under different durability test conditions. After water soaking, dry–wet cycle, acid/alkali corrosion, the retention rates of tensile and shear properties of PCJS can achieve 80%. After UV aging and low temperature treatment, the tensile and shear properties of PCJS are improved. In addition to gasoline corrosion and high temperature treatment, PCJS maintains a continuous polymer film structure in other durability test conditions.
3. After gasoline corrosion, the retention rates of tensile and shear properties of PCJS exhibit greater than 75%. After high temperature treatment, the retention rates of tensile and shear properties of PCJS exhibit greater than 60%. The pore size of PCJS increases and pore structure deteriorates after gasoline corrosion and high temperature treatment.

The durability of PCJS under different service conditions was studied, but the durability of PCJS under multiple service conditions was not involved. In addition, the microstructure of PCJS was only studied by SEM and MIP tests, and it lacked the analysis of reaction products by XRD or FTIR tests. At present, the relevant standards of joint sealant involve its preparation and property testing, and it is necessary to develop the standards for the durability of joint sealant. On the whole, PCJS shows excellent durability and can be applied to joint engineering of cement concrete pavement.

## Data Availability

The data that support the findings of this study are available on request from the corresponding author.
